# Sex Differences in the Association of Sibship Size and Position in Sibship with Lipid Profile during Adolescence: A Cross-Sectional Study

**DOI:** 10.1155/2022/8727922

**Published:** 2022-09-27

**Authors:** Ali H. Ziyab, Mohammad Almari, Anwar Mohammad, Abdullah Al-Taiar, Wilfried Karmaus

**Affiliations:** ^1^Department of Community Medicine and Behavioral Sciences, Faculty of Medicine, Kuwait University, Safat, Kuwait; ^2^Department of Health Policy and Management, Faculty of Public Health, Kuwait University, Safat, Kuwait; ^3^Biochemistry and Molecular Biology Department, Research Division, Dasman Diabetes Institute, Kuwait, Kuwait; ^4^School of Community and Environmental Health, College of Health Sciences, Old Dominion University, Norfolk, VA, USA; ^5^Division of Epidemiology, Biostatistics, and Environmental Health, School of Public Health, University of Memphis, Memphis, TN, USA

## Abstract

**Background:**

Epidemiologic studies have reported associations of sibship size and position of the child in the sibship with multiple health outcomes, including adiposity and diabetes. However, little is known about sibling effects on lipids. Hence, this study sought to evaluate associations of the number of total, older, and younger siblings with lipid profile among adolescents.

**Methods:**

In a cross-sectional study among high school students aged 14 to 19 years, lipid levels were measured in capillary blood. Parents reported the number of siblings (total, older, and younger). Geometric means of lipids were calculated, and linear regression was used to estimate the ratio of geometric means (RoGM) and 95% confidence intervals (CI). Analyses were sex stratified.

**Results:**

Of the total study sample (*n* = 1,584), 758 (47.9%) were boys and 826 (52.1%) were girls, with median age of 16.0 years. Total cholesterol (TC) was lower by 8% (adjusted-RoGM = 0.92, 95% CI: 0.88–0.96) among boys with ≥3 older siblings compared to those with no older siblings. Similarly, boys with ≥3 younger sibling compared to those with no younger siblings had reduced TC by 7% (adjusted-RoGM = 0.93, 0.87–0.99). Moreover, an increased number of total siblings (≥4 vs. 0/1: adjusted-RoGM = 0.80, 0.67–97) and older siblings (≥3 vs. 0: adjusted-RoGM = 0.90, 0.82–0.98) were associated with reduced low-density lipoprotein cholesterol (LDL-C) among boys. Similarly, lower levels of triglycerides (TG) were seen among boys with ≥3 older siblings compared to those with no older siblings (adjusted-RoGM = 0.87, 0.78–0.96). A higher number of younger siblings was associated with increased high-density lipoprotein cholesterol (HDL-C) among boys (≥3 vs. 0: adjusted-RoGM = 1.08, 1.01–1.17). Sibship characteristics were not associated with lipids among girls.

**Conclusions:**

Increased number of total, older, and younger siblings were associated with favorable lipid profiles among adolescent boys, but not girls. Mechanisms underlying these associations need further investigations.

## 1. Introduction

The “sibling effect” concept refers to the epidemiologic observation of the associations of sibship size and/or the position of the child in the sibship with various health outcomes. Several characterizations are used to describe the sibling effect, including the number of total siblings (sibship size), number of older siblings (i.e., birth order − 1), number of younger siblings, number of brothers or sisters, first-born, and only-child [1]. Studies from different research fields have investigated the sibling effect on various health outcomes and indicators among children. For example, in 1986, Golding and Peters reported inverse associations between the number of siblings and eczema and hay fever among children aged 5 years [2], and these observations were subsequently corroborated [3–5]. Some studies have further showed that position in the sibship, i.e., number of older or younger siblings, is a better predictor of allergies than sibship size [6–8]. Moreover, negative associations were reported between sibship size and position in the sibship with growth [9], adiposity [10, 11], diabetes [12], and blood pressure [13]. On the contrary, a study has shown positive association between sibship characteristics and psoriasis [14]. However, mechanisms underlying these associations are unclear, with hypotheses implicating prenatal exposures leading to intrauterine programming and early life factors [15, 16].

An effect of siblings on lipid profile in childhood has been scarcely explored, with inconsistent results in prior studies. Ayyavoo et al. reported that the lipid profile was not significantly different between first-born and later-born children aged 4 to 11 years [17]. Similarly, Savage et al. demonstrated similar lipid profile in first-born and later-born children aged 3 to 10 years [18]. In contrast, an unfavorable lipid profile in firstborns compared to laterborns was reported among male Brazilian adolescents aged 17 to 19 years [19]. A study based on a large sample of Chinese children aged 6 to 17 years has shown that only-children had an abnormal lipid profile compared to children with siblings [20]. These results suggest a possible influence of siblings on the lipid profile, which is important, since lipid abnormalities in childhood/adolescence track into adulthood and are indicators of future cardiovascular diseases [21–23].

Previous studies have investigated the sibling effect on lipid profile using dichotomous approaches (e.g., firstborns versus laterborns or only-children versus children with siblings) [17–20], which did not allow to investigate a dose-response relationship between lipid profile and sibship size or position of the child in the sibship. Such an approach will help to better uncover the true relationship between sibling effect and lipid profile and to understand the potential mode of action of siblings. Furthermore, none of the previous studies was in Middle Eastern settings, and the association between sibling effect and lipid profile remains unknown in this region. To this end, the current study sought to assess associations between the number of total siblings, number of older siblings, and number of younger siblings with lipid profile among adolescents in Kuwait. We have stratified our analysis by sex based on the existing evidence that males and females exhibit differential lipid profile and metabolic syndrome risk [24, 25].

## 2. Methods

### 2.1. Study Setting, Design, and Participants

This cross-sectional study enrolled high school students (*n* = 1959; grades 10, 11, and 12; aged 14–19 years) attending public schools across the state of Kuwait between September and December 2017. A stratified random sampling method was used in selecting schools and students as detailed by Almari et al. [26]. In the current analysis, we excluded participants who reported a history of doctor-diagnosed diabetes (*n* = 97), participants with undiagnosed diabetes (i.e., no prior history of doctor-diagnosed diabetes and measured HbA1c (glycated hemoglobin) ≥6.5% (48 mmol/mol); *n* = 17), and participants with missing information for all lipid variables (i.e., total cholesterol (TC), low-density lipoprotein cholesterol (LDL-C), high-density lipoprotein cholesterol (HDL-C), and triglycerides (TG); *n* = 261). Of the total enrolled study sample, 80.9% (1,584/1,959) satisfied our inclusion criteria. This study was approved by the Health Sciences Center Ethical Committee at Kuwait University (no. VDR/EC/3067). The study was conducted in accordance with principles and guidelines of the Declaration of Helsinki for medical research involving human subjects. Written informed consent was obtained from parents or legal guardians to enroll study participants. Subsequently, self-administered questionnaires were completed by parents and students.

### 2.2. Biochemical Analyses and Prediabetes Definition

The point-of-care Cobas b 101 system (Roche Diagnostics, Mannheim, Germany) was used to measure HbA1c and lipid profile in nonfasting capillary blood. Two types of test discs were used, which are as follows: the Hb1Ac test disc and the lipid panel test disc that quantitatively determined TC, HDL-C, and TG and provided a calculated value for LDL-C using the Friedewald formula when the concentration of TG was <4.52 mmol/L: LDL = TC–HDL–TG/2.22 [27]. According to the manufacturer's performance evaluation report [28], the Cobas b 101 system met the national glycohemoglobin standardization program (NGSP) acceptance criteria for measuring HbA1c [29] and met the national cholesterol education program (NCEP) guidelines for measuring lipids [30]. Prediabetes was defined according to the American Diabetes Association (ADA) criteria: 5.7% ≤ HbA1c ≤ 6.4% (39–47 mmol/mol) [31].

### 2.3. Anthropometric Measurements

Height was measured to the nearest 0.1 centimeter (cm) using a stadiometer while weight was measured to the nearest 0.1 kilogram (kg) using a digital scale. Both height and weight were measured without shoes and in light clothing in a standardized manner. Body mass index (BMI) was calculated as weight in kilograms divided by height in meters squared (kg/m^2^). Since BMI, a measure of general adiposity, changes markedly with growth in children and adolescents, we estimated BMI-for-age z-scores (standard deviation; SD) using the World Health Organization (WHO) growth reference for those aged between 5 and 19 years [32]. BMI-for-age was categorized as follows: thinness: <−2 SD, normal: −2 to 1 SD, overweight: >1 to 2 SD, and obese: >2 SD [32].

### 2.4. Ascertainment of Sibship Characteristics and Covariates

Information on the child's number of total, older, and younger siblings born to the same mother, mode of delivery, ever breastfed as an infant, maternal and paternal education level, household secondhand smoke exposure, and maternal and paternal history of doctor-diagnosed diabetes was collected through a self-administered questionnaire that was completed by parents as described in details previously [26, 33]. The child was considered to have a parental history of diabetes if the mother and/or father ever reported a history of doctor-diagnosed diabetes. Exposure to household secondhand smoke was ascertained by an affirmative response by parents to the question, “Does anyone smoke cigarettes or water-pipe inside the house?” Moreover, participants self-reported their current smoking status by answering the following question: “have you smoked at least one combustible cigarette in the past 30 days?” The frequency of engaging in vigorous physical activity was assessed by the subsequent question, which was answered by the students. “How many times a week do you engage in vigorous physical activity long enough to make you breathe hard?”

### 2.5. Statistical Analysis

Analyses were conducted using SAS 9.4 (SAS Institute, Cary, NC, USA). The statistical significance level was set to *α* = 0.05 for all association analyses. Descriptive analyses were conducted to calculate frequencies and proportions of categorical variables in the total sample and after stratification by sex. To account for the skewed distribution of lipid variables, geometric means were estimated by log_10_-transformation of the data and subsequently taking the antilog of the calculated means on the transformed scale.

The number of total siblings was categorized into four groups (0 and 1, 2, 3, and ≥4 siblings). We combined the 0 and 1 sibling categories as only one child had zero siblings. The numbers of older and younger siblings were analyzed using the following categories: 0, 1, 2, and ≥3. The associations of the number of total, older, and younger siblings (exposure variables) with log_10_-transformed lipid variables (outcome variables) were evaluated using multiple linear regression models while adjusting for the effects of age, BMI-for-age *z*-scores, mode of delivery, breastfeeding status, smoking status, frequency of vigorous physical activity per week, exposure to household secondhand smoke, prediabetes status, parental history of diabetes, maternal education level, and paternal education level. When assessing the association between the number of older siblings and lipid variables, the number of younger siblings was included as a covariate in the regression model, and vice versa. Given that boys and girls are characterized by different lipid profiles [24, 25], our analysis was stratified by sex and separate models were evaluated for boys and girls. Using the total study sample, statistical interactions on multiplicative scale between sex and sibship characteristics were evaluated by including product terms (sex × total/older/younger number of siblings) in regression models. Given that statistical power to detect higher-order terms is usually limited in epidemiologic studies [34, 35], the interaction term *P*-value (*P*_interaction_) < 0.2 was considered a “possible” statistical suggestion for interaction (effect modification). Given that we regressed log_10_-transformed lipid values, taking the antilog of the linear regression coefficients (*β*) yields adjusted ratio of geometric means (RoGM), but not the difference between geometric means [36]. Hence, the related 95% confidence intervals (CIs) represent limits for RoGM with a null value of 1.

## 3. Results

### 3.1. Characteristics of the Study Sample

In total, 1959 high school students were enrolled in the current study (899 boys and 1060 girls). Of the total enrolled participants, 375 subjects were excluded from the analytical study sample (*n* = 1584) as detailed in the methods section. Of the total analytical sample, 758 (47.9%) were boys and 826 (52.1%) were girls (Table 1). The median age of the study participants was 16.0 years (5^th^, 95^th^ percentile: 14.0, 18.0 years). Based on BMI-for-age categories, obesity was more prevalent in boys (38.6%, 293/758) than in girls (24.4%, 201/825). Prediabetes affected 34.3% (543/1,584) of participants, with no difference between boys and girls (Table 1). Only one participant had no sibling. Most study participants had four or more (≥4) siblings (76.7%, 1172/1528), three or more (≥3) older siblings (37.8%, 593/1570), and ≥3 younger siblings (56.5%, 859/1519; Table 1).

### 3.2. Associations between Sibship Characteristics and Lipid Levels

The number of total siblings was not associated with TC levels in both sexes (Table 2). However, TC levels were lower by 8% (adjusted-RoGM = 0.92, 95% CI: 0.88–0.96) among boys who had ≥3 older siblings compared to those who had no older siblings. Similarly, a reduction in TC levels by 7% (adjusted-RoGM = 0.93, 95% CI: 0.87–0.99) was seen among boys with ≥3 younger siblings compared to those with no younger siblings. Among girls, the number of older and younger siblings were not associated with TC levels (Table 2). Tests for sex-related interaction showed that the effect of older siblings on TC levels was different among boys and girls (*P*_interaction_=0.021). However, there was no statistical evidence for sex-related differential effect of younger siblings on TC levels (*P*_interaction_=0.731; Table 2).

Among boys, compared to adolescents with no or one sibling, those in large sibships had lower LDL-C levels (3 siblings: 20% reduction (adjusted-RoGM = 0.80, 95% CI: 0.66–98); ≥4 siblings: 20% reduction (adjusted-RoGM = 0.80, 95% CI: 0.67–97) Table 3). Similarly, boys with ≥3 older siblings had reduced LDL-C levels by 10% (adjusted-RoGM = 0.90, 95% CI: 0.82–0.98) compared to those with no older siblings, whereas the number of younger siblings was not associated with LDL-C levels in boys. Among girls, sibship size and position in the sibship were not associated with LDL-C levels (Table 3). A possible statistical suggestion of a sex-related interaction was observed for total siblings effect (*P*_interaction_=0.149) and older siblings effect (*P*_interaction_=0.071; Table 3) on LDL-C levels. In addition to statistical testing, the direction of the point estimates (i.e., RoGM) further support the possible presence of effect modification by sex.

Levels of HDL-C were higher by 10% (adjusted-RoGM = 1.10, 95% CI: 1.02–1.19) and 8% (adjusted-RoGM = 1.08, 95% CI: 1.01–1.17) among boys who had 2 and ≥3 younger siblings compared to those with no younger siblings, respectively (Table 4). The number of total and older siblings were not associated with HDL-C levels in boys. Moreover, HDL-C levels were not affected by the number of total, older, and younger siblings in girls (Table 4). The observed sex-specific effect of younger siblings on HDL-C levels in boys and the absence of such association in girls is supported by a possible statistical interaction (*P*_interaction_=0.094) and by the direction of effects, whilst in males, more younger siblings were associated with higher HDL-C, and more younger siblings in females were not associated with HDL-C.

Boys with older siblings had lower TG levels compared to those with no older siblings (Table 5). For instance, boys with 2 and ≥3 older siblings had reduced TG levels by 17% (adjusted-RoGM = 0.83, 95% CI: 0.74–0.94) and 13% (adjusted-RoGM = 0.87, 95% CI: 0.78–0.96), respectively, compared to those with no older siblings. However, the number of total and younger siblings were not associated to TG levels in boys. Among girls, the sibship size and position in the sibship were not associated with TG levels (Table 5). A possible statistical suggestion of a sex-related interaction was observed for older siblings effect on TG levels (*P*_interaction_=0.158; Table 5).

## 4. Discussion

This study examined associations between lipid profile and both sibship size and the position of the child in the sibship among a school-based sample of adolescents in Middle Eastern setting. Overall, the results of this report suggest that higher number of total, older, and younger siblings to be favorably associated with lipid levels among boys. However, lipid levels among girls were not influenced by sibship characteristics. After adjusting for multiple potential confounders, we found that having both ≥3 older and younger siblings was associated with reduced TC levels among boys. Moreover, boys from large sibships (3 and ≥4 total siblings) and those with ≥3 older siblings had reduced LDL-C levels. Similarly, lower levels of TG were seen among boys with 2 and ≥3 older siblings. A higher number of younger siblings was also associated with favorable (i.e., increased) HDL-C levels among boys. The findings of this report highlight new aspects related to the effects of sibship characteristics on lipid profile during adolescence.

Few prior studies have investigated the associations between sibship characteristics and lipid profile and reported mixed results [17–20]. Two studies have shown that lipid profile was not significantly different when comparing firstborns to laterborns [17, 18]. However, a study among Brazilian male adolescents [19] and a study among Chinese children [20] have demonstrated that firstborns and only-children, respectively, had altered lipid levels. Our study extends prior observations by further investigating the dose-response effects of total, older, and younger siblings on lipid profile while stratifying by sex. Our analytical approach of investigating the ordinal number of siblings rather than the dichotomous categorization showed that effects of siblings on lipid profile become apparent in larger sibships. For instance, TC levels were lower among boys with ≥3 older or ≥3 younger siblings compared to those with no older or no younger siblings (Table 2). Moreover, among male participants, having 3 or ≥4 total siblings as well as ≥3 older siblings associated with reduced LDL-C levels (Table 3). Similar observations of sibling effects were seen for HDL-C and TG, where higher number of younger and older siblings, respectively, associated with altered lipid levels in boys (Tables 4 and 5). Whereas, in general, lipid profiles of adolescents who had 1 or 2 older as well as 1 or 2 younger siblings did not differ from those with no older or no younger siblings. These observations indicate that the sibling effect on lipid profile might be missed when comparing first-borns to later-borns. Therefore, our findings provide a novel contribution and demonstrate the ordinal effect of siblings due to the large sibship sizes in families in Kuwait (76.7% reported having ≥4 siblings).

Similarly, the sex-specific associations between sibship characteristics and lipid profile observed in this report are novel. Using sex-stratified analysis, a previous study among Chinese children showed unfavorable lipid profile to be associated with being only-child among both sexes [20], which contradicts our observation of sex-specific effects. Given that sex-specific sibship effects have been rarely investigated, future studies are needed to corroborate our findings of sex-specific associations. Our motive to conduct sex-specific analysis was based on the fact that males and females exhibit differential lipid profile and metabolic syndrome risk, and these sex-dimorphisms are mediated by the effects of sex hormones [24, 25, 37, 38]. Moreover, accumulating evidence suggest that prenatal and postnatal factors might influence disease risk in a sex-specific manner [39, 40]. Hence, reporting association separately for males and females might provide better insights into the link between sibling effects and dyslipidemia and metabolic risk.

The mechanisms and factors mediating the effects of sibship size and the position of the child in the sibship on health outcomes need to be further explored. Having older siblings may induce prenatal programming and/or resemble postnatal effects. However, the effects associated with having younger siblings are likely to be because of postnatal factors. For instance, children with more siblings (older or younger) compared to those with a few/no siblings have greater opportunities to be involved in physical activities [41]. Therefore, the observed protective effects of larger sibships could be because of such social and environmental factors. However, sibship characteristics were not associated with the frequency of vigorous physical activity in our study sample (see online Supplementary Table S1). A competing hypothesis suggests that in utero programming could explain the observed effects of older siblings [4, 7]. Programming towards endocrine and metabolic dysfunction has been linked to intrauterine exposures (e.g., gestational diabetes, smoking, maternal diet, and obesity) and postnatal factors (feeding practices during infancy, early excess weight gain, and exposure to endocrine-disrupting chemicals) [15, 16]. In our analysis, the effects of older siblings on lipid levels were more pronounced, to some extent, than the effects of younger siblings on lipid levels. Apart from the possible intrauterine priming, children with many older siblings could be an indicator of more experienced parents that ensure a healthy childhood environment (e.g., healthy diet). Nevertheless, future studies exploring mechanisms underlying the sibling effects are needed.

Enrolling a large sample of adolescents based on a random school-based sampling is a major strength of our study. The accuracy of the point-of-care systems in measuring lipid profile has been speculated. Nonetheless, prior studies have shown that the Cobas b 101 point-of-care system (used in this study) provides valid and reliable lipid measurements and meets the NCEP guidelines [28, 42]. Therefore, bias because of the random effects of measurement error, if any, will likely underestimate the magnitude of the reported associations. Moreover, although measuring lipid profiles in nonfasting capillary blood is a potential limitation in our study, it has been shown that nonfasting state does not affect the lipid profile assessment [43]. A prior study among children showed negligible differences between lipid profiles measured in fasting and nonfasting blood samples [44]. Using the Friedewald formula to estimate LDL-C instead of direct measurement is a further limitation to our study. Moreover, the lack of information on birth weight, maternal age at birth, gestational weight gain, gestational diabetes and hypertension, and participants' diet is a further limitation to our study. It needs to be addressed in future investigations. Age and adiposity have been shown to alter lipid levels among adolescents [45, 46]. Nonetheless, our reported associations were independent of the effects of age (surrogate variable for pubertal stage) and adiposity as we have adjusted for their effects in the regression models. We initially have enrolled a total of 1959 subjects. However, 375 subjects did not meet the inclusion criteria of the present study (97 subjects were classified to have prediabetes, 17 subjects had undiagnosed diabetes, and 261 subjects did not have their lipids measured because of the unavailability of the lipid panel test discs). Nonetheless, we have shown in a previous study that the analyzed sample (*n* = 1584) did not differ from the total enrolled sample (*n* = 1959) [33], and hence, selection bias is not a major source of concern. Moreover, we acknowledge the fact that our study might have been statistically underpowered to detect statistically significant interaction terms at significance level of 0.05. Hence, to avoid false negative results and to mistakenly indicate the absence of sex differences (i.e., no effect modification by sex), we have applied a less stringent significance level of 0.2 to indicate a “possible” statistical suggestion for interaction [34, 47]. A strength of our analyses is that because of a higher number of children in Kuwaiti families, we were able to disentangle the effects of older and younger siblings, which has not been done in the previous studies [17–20]. In addition, we adjusted for the concurrent smoke exposure of the participant (active and passive) and their physical activity. Both factors could have otherwise contributed to different lipid levels.

## 5. Conclusions

The findings of this report suggest that the number of siblings is associated with lipid profile among male adolescents. Specifically, a higher number of older siblings was associated with reduced levels of TC, LDL-C, and TG among boys. A higher number of younger siblings was associated with reduced TC levels and increased HDL-C levels among boys. Among female adolescents, the number of total, older, and younger siblings did not influence lipid levels. The observed sex-specific effects of sibship characteristics on lipids is novel and needs further corroboration. The sibling effects implicate the influence of prenatal and postnatal programming. However, the question of which causal factors explain the sibling effects remains unanswered. On the other hand, we have to consider that lipid levels in adolescence are predictive of adult cardiovascular diseases. Thus, a better understanding of the development of these biomarkers is essential.

## Figures and Tables

**Table 1:** Characteristics of the study participants in the total sample and stratified by sex.

Variables	Total (*n* = 1,584)	Boys (*n* = 758)	Girls (*n* = 826)
Age groups (years), % (*n*)			
≤15	37.0 (582)	35.5 (269)	38.3 (313)
16	29.5 (465)	29.6 (224)	29.4 (241)
≥17	33.5 (528)	34.9 (264)	32.3 (264)

Missing, (*n*)	(9)	(1)	(8)

Mode of delivery, % (*n*)			
Vaginal	85.3 (1323)	84.1 (619)	86.4 (704)
Cesarean section	14.7 (228)	15.9 (117)	13.6 (111)

Missing, (n)	(33)	(22)	(11)

Ever breastfed, % (*n*)			
Yes	82.9 (1290)	82.7 (612)	83.0 (678)

Missing, (*n*)	(27)	(18)	(9)

Smoking status, % (*n*)			
Current (past 30-day)	10.7 (169)	21.9 (165)	0.5 (4)

Missing, (*n*)	(10)	(4)	(6)

Household secondhand smoke, % (*n*)			
Yes	50.5 (791)	50.4 (377)	50.7 (414)

Missing, (*n*)	(19)	(10)	(9)

BMI-for-age groups, % (*n*)			
Thinness (<−2 SD)	2.3 (36)	2.8 (21)	1.8 (15)
Normal (−2 to 1 SD)	43.9 (692)	36.8 (279)	50.5 (413)
Overweight (>1 to 2 SD)	22.6 (356)	21.8 (165)	23.4 (191)
Obesity (>2 SD)	31.2 (491)	38.6 (292)	24.3 (199)

Missing, (*n*)	(9)	(1)	(8)

Vigorous physical activity per week, % (*n*)			
Never or occasionally	31.7 (500)	25.7 (194)	37.1 (306)
Once or twice a week	47.6 (751)	46.6 (351)	48.6 (400)
Three or four times a week	12.2 (192)	15.0 (113)	9.6 (79)
Five or more times a week	8.5 (135)	12.7 (96)	4.7 (39)

Missing, (n)	(6)	(4)	(2)

Prediabetes, % (*n*)			
Yes (5.7 ≤ HbA1c % ≤ 6.4)	34.3 (543)	34.8 (264)	33.8 (279)

Parental history of diabetes^*∗*^, % (*n*)			
Yes	41.6 (650)	39.3 (291)	43.7 (359)

Missing, (*n*)	(22)	(17)	(5)

Total siblings, % (*n*)			
0	0.1 (1)	N/A	0.1 (1)
1	2.7 (42)	2.9 (21)	2.6 (21)
2	6.3 (96)	7.2 (52)	5.5 (44)
3	14.2 (217)	16.6 (119)	12.1 (98)
≥4	76.7 (1172)	73.3 (527)	79.7 (645)

Missing, (*n*)	(56)	(39)	(17)

Older siblings, % (*n*)			
0	24.2 (380)	25.0 (187)	23.5 (193)
1	19.4 (304)	19.5 (146)	19.2 (158)
2	18.6 (293)	18.5 (139)	18.8 (154)
≥3	37.8 (593)	37.0 (277)	38.5 (316)

Missing, (*n*)	(14)	(9)	(5)

Younger siblings, % (*n*)			
0	7.6 (115)	8.0 (57)	7.2 (58)
1	17.3 (263)	16.5 (118)	18.1 (145)
2	18.6 (282)	20.1 (144)	17.2 (138)
≥3	56.5 (859)	55.5 (397)	57.5 (462)

Missing, (*n*)	(65)	(42)	(23)

BMI: body mass index; SD: standard deviation; N/A: not applicable; HbA1c: glycated hemoglobin. ^*∗*^Maternal and/or paternal history of doctor-diagnosed diabetes.

**Table 2:** Geometric means and adjusted ratio of geometric means of total cholesterol according to the total number of siblings, number of older siblings, and number of younger siblings stratified by sex.

	Outcome variable: total cholesterol						Sex-sibling*P*_interaction_^#^
	Boys			Girls			
	*n*	Geometric mean (mmol/L)	Ratio of geometric means (95% CI)^‡^	*n*	Geometric mean (mmol/L)	Ratio of geometric means (95% CI)^‡^	
Total siblings							0.535
0 and 1^†^	21	3.38	1.00 (reference)	22	3.85	1.00 (reference)	
2	52	3.54	1.05 (0.94–1.16)	44	3.80	0.99 (0.90–1.08)	
3	119	3.46	1.02 (0.93–1.13)	98	3.72	0.97 (0.89–1.05)	
≥4	527	3.24	0.96 (0.87–1.05)	645	3.62	0.94 (0.87–1.02)	

Older siblings							0.021
0	187	3.47	1.00 (reference)	193	3.78	1.00 (reference)	
1	146	3.48	1.00 (0.96–1.05)	158	3.65	0.97 (0.93–1.00)	
2	139	3.35	0.97 (0.92–1.01)	154	3.69	0.98 (0.94–1.02)	
≥3	277	3.19	0.92 (0.88–0.96)^*∗*^	316	3.69	0.98 (0.94–1.01)	

Younger siblings							0.731
0	57	3.51	1.00 (reference)	58	3.74	1.00 (reference)	
1	118	3.35	0.96 (0.89–1.02)	145	3.76	1.01 (0.95–1.06)	
2	144	3.36	0.96 (0.90–1.02)	138	3.68	0.98 (0.93–1.04)	
≥3	397	3.26	0.93 (0.87–0.99)^§^	462	3.62	0.97 (0.92–1.02)	

CI: confidence interval. ^†^Since only one child had zero siblings, we combined the no siblings and one sibling groups: reported as: 0 and 1. ^‡^Adjusted for age, body mass index-for-age, mode of birth, breastfeeding status, smoking status, frequency of vigorous physical activity per week, exposure to household secondhand smoke, prediabetes status, parental history of diabetes, maternal education level, and paternal education level. Additionally, the ratios of geometric means of older siblings were simultaneously adjusted for younger siblings, and the ratios of geometric means of younger siblings were simultaneously adjusted for older siblings. ^§^*P*-value <0.05; ^*∗*^*P*-value <0.01. ^#^*P*-value evaluating statistical interaction on multiplicative scale between sex and number of total, older, and younger siblings in the total study sample. An interaction *P*-value <0.2 was considered to be a “possible” statistical suggestion for interaction.

**Table 3:** Geometric means (GM) and adjusted ratio of geometric means of low-density lipoprotein cholesterol according to the total number of siblings, number of older siblings, and number of younger siblings stratified by sex.

	Outcome variable: low-density lipoprotein cholesterol						Sex-sibling *P*_interaction_^#^
	Boys			Girls			
	*n*	Geometric mean (mmol/L)	Ratio of geometric means (95% CI)^‡^	*n*	Geometric mean (mmol/L)	Ratio of geometric means (95% CI)^‡^	
Total siblings							0.149
0 and 1^†^	21	1.96	1.00 (reference)	21	1.91	1.00 (reference)	
2	52	1.74	0.89 (0.72–1.10)	44	1.80	0.95 (0.77–1.16)	
3	116	1.57	0.80 (0.66–0.98)^§^	97	1.87	0.98 (0.82–1.18)	
≥4	517	1.57	0.80 (0.67–0.97)^§^	638	1.80	0.95 (0.80–1.12)	

Older siblings							0.071
0	182	1.68	1.00 (reference)	188	1.86	1.00 (reference)	
1	144	1.67	1.00 (0.91–1.10)	158	1.82	0.98 (0.90–1.06)	
2	138	1.65	0.98 (0.89–1.08)	154	1.88	1.01 (0.93–1.10)	
≥3	272	1.51	0.90 (0.82–0.98)^*∗*^	312	1.87	1.00 (0.93–1.09)	

Younger siblings							0.804
0	57	1.62	1.00 (reference)	58	1.74	1.00 (reference)	
1	118	1.68	1.04 (0.91–1.18)	142	1.92	1.10 (0.98–1.24)	
2	142	1.63	1.01 (0.88–1.15)	137	1.94	1.12 (0.99–1.26)	
≥3	386	1.58	0.97 (0.86–1.10)	457	1.82	1.05 (0.94–1.17)	

CI: confidence interval. ^†^Since only one child had zero siblings, we combined the no siblings and one sibling groups: reported as: 0 and 1. ^‡^Adjusted for age, body mass index-for-age, mode of birth, breastfeeding status, smoking status, frequency of vigorous physical activity per week, exposure to household secondhand smoke, prediabetes status, parental history of diabetes, maternal education level, and paternal education level. Additionally, ratios of geometric means of older siblings were simultaneously adjusted for younger siblings, and ratios of geometric means of younger siblings were simultaneously adjusted for older siblings. ^§^*P*-value <0.05; ^*∗*^*P*-value <0.01. ^#^*P*-value evaluating statistical interaction on multiplicative scale between sex and number of total, older, and younger siblings in the total study sample. An interaction *P*-value <0.2 was considered to be a “possible” statistical suggestion for interaction.

**Table 4:** Geometric means and adjusted ratio of geometric means of high-density lipoprotein cholesterol according to the total number of siblings, number of older siblings, and number of younger siblings stratified by sex.

	Outcome variable: High-density lipoprotein cholesterol						Sex-sibling *P*_interaction_^#^
	Boys			Girls			
	*n*	Geometric mean (mmol/L)	Ratio of geometric means (95% CI)^‡^	*n*	Geometric mean (mmol/L)	Ratio of geometric means (95% CI)^‡^	
Total siblings							0.203
0 and 1^†^	21	1.08	1.00 (reference)	22	1.32	1.00 (reference)	
2	52	1.23	1.14 (0.99–1.30)	44	1.29	0.98 (0.87–1.10)	
3	119	1.14	1.06 (0.93–1.20)	98	1.33	1.01 (0.91–1.13)	
≥4	527	1.11	1.02 (0.91–1.15)	644	1.27	0.96 (0.87–1.06)	

Older siblings							0.625
0	187	1.12	1.00 (reference)	192	1.27	1.00 (reference)	
1	146	1.10	0.98 (0.92–1.04)	158	1.25	0.99 (0.94–1.04)	
2	139	1.13	1.01 (0.95–1.07)	154	1.26	1.00 (0.95–1.05)	
≥3	277	1.09	0.97 (0.92–1.03)	316	1.29	1.02 (0.97–1.07)	

Younger siblings							0.094
0	57	1.04	1.00 (reference)	58	1.29	1.00 (reference)	
1	118	1.12	1.07 (0.99–1.17)	145	1.28	1.00 (0.93–1.07)	
2	144	1.14	1.10 (1.02–1.19)^§^	138	1.24	0.97 (0.90–1.04)	
≥3	397	1.13	1.08 (1.01–1.17)^§^	461	1.25	0.98 (0.91–1.04)	

CI: confidence interval. ^†^Since only one child had zero siblings, we combined the no siblings and one sibling groups: reported as: 0 and 1. ^‡^Adjusted for age, body mass index-for-age, mode of birth, breastfeeding status, smoking status, frequency of vigorous physical activity per week, exposure to household secondhand smoke, prediabetes status, parental history of diabetes, maternal education level, and paternal education level. Additionally, ratios of geometric means of older siblings were simultaneously adjusted for younger siblings, and ratios of geometric means of younger siblings were simultaneously adjusted for older siblings. ^§^*P*-value <0.05. ^#^*P*-value evaluating statistical interaction on multiplicative scale between sex and number of total, older, and younger siblings in the total study sample. An interaction *P*-value <0.2 was considered to be a “possible” statistical suggestion for interaction.

**Table 5:** Geometric means and adjusted ratio of geometric means of triglycerides according to the total number of siblings, number of older siblings, and number of younger siblings stratified by sex.

	Outcome variable: Triglycerides						Sex-sibling *P*_interaction_^#^
	Boys			Girls			
	*n*	Geometric mean (mmol/L)	Ratio of geometric means (95% CI)^‡^	*n*	Geometric mean (mmol/L)	Ratio of geometric means (95% CI)^‡^	
Total siblings							0.127
0 and 1^†^	21	1.08	1.00 (reference)	22	1.07	1.00 (reference)	
2	52	1.39	1.29 (0.99–1.66)	44	0.95	0.88 (0.68–1.15)	
3	118	1.31	1.22 (0.96–1.54)	98	1.08	1.00 (0.79–1.27)	
≥4	524	1.24	1.15 (0.92–1.43)	642	1.02	0.95 (0.77–1.18)	

Older siblings							0.158
0	186	1.41	1.00 (reference)	193	1.06	1.00 (reference)	
1	145	1.32	0.94 (0.84–1.05)	156	1.04	0.98 (0.88–1.10)	
2	139	1.17	0.83 (0.74–0.94)^*∗*^	153	1.05	0.99 (0.89–1.11)	
≥3	275	1.22	0.87 (0.78–0.96)^*∗*^	316	1.02	0.96 (0.87–1.06)	

Younger siblings							0.893
0	57	1.36	1.00 (reference)	58	1.09	1.00 (reference)	
1	118	1.28	0.94 (0.80–1.10)	145	1.06	0.97 (0.83–1.14)	
2	144	1.22	0.90 (0.77–1.05)	138	1.00	0.92 (0.78–1.08)	
≥3	393	1.24	0.91 (0.78–1.05)	459	1.03	0.94 (0.81–1.09)	

CI: confidence interval. ^†^Since only one child had zero siblings, we combined the no siblings and one sibling groups: reported as: 0 and 1. ^‡^Adjusted for age, body mass index-for-age, mode of birth, breastfeeding status, smoking status, frequency of vigorous physical activity per week, exposure to household secondhand smoke, prediabetes status, parental history of diabetes, maternal education level, and paternal education level. Additionally, ratios of geometric means of older siblings were simultaneously adjusted for younger siblings, and ratios of geometric means of younger siblings were simultaneously adjusted for older siblings. ^*∗*^*P*-value <0.01. ^#^*P*-value evaluating statistical interaction on multiplicative scale between sex and number of total, older, and younger siblings in the total study sample. An interaction *P*-value <0.2 was considered to be a “possible” statistical suggestion for interaction.

## Data Availability

The datasets used and/or analyzed during the current study can be obtained from the corresponding author on reasonable request.

## Supplementary Materials

Supplementary Materials Supplementary Table: table S1. Associations of total number of siblings, number of older siblings, and number of younger siblings with frequency of vigorous physical activity per week stratified by sex.
