# Supplementary material for: Sex Differences in the Association of Sibship Size and Position in Sibship with Lipid Profile during Adolescence: A Cross-Sectional Study
Source: Int J Endocrinol. 2022 Sep 27;2022:8727922. doi: 10.1155/2022/8727922 (PMC9532113; doi:10.1155/2022/8727922)
Supplement: Supplementary Materials — Supplementary Table: table S1. Associations of total number of siblings, number of older siblings, and number of younger siblings with frequency of vigorous physical activity per week stratified by sex. [file 8727922.f1.pdf]

Table S1. Associations of total number of siblings, number of older siblings, and number of younger siblings with frequency of vigorous physical activity per week stratified by sex

Vigorous physical activity per week	Total siblings					Older siblings					Younger siblings				
	**0 & 1**	**2**	**3**	**≥4**	**P***	**0**	**1**	**2**	**≥3**	**P***	**0**	**1**	**2**	**≥3**	**P***
**Boys**	**(n=21)**	**(n=52)**	**(n=118)**	**(n=524)**		**(n=187)**	**(n=144)**	**(n=139)**	**(n=275)**		**(n=55)**	**(n=118)**	**(n=143)**	**(n=396)**	
**Never/occasionally**	23.8 (5)	15.4 (8)	20.3 (24)	28.4 (149)	0.336	26.7 (50)	21.5 (31)	23.0 (32)	28.4 (78)	0.397	23.6 (13)	22.0 (26)	24.5 (35)	28.0 (111)	0.949
**Once or twice**	42.9 (9)	53.9 (28)	50.0 (59)	44.9 (235)		44.4 (83)	52.8 (76)	46.0 (64)	45.5 (125)		45.5 (25)	50.0 (59)	47.6 (68)	44.7 (177)	
**Three or four times**	19.1 (4)	23.1 (12)	15.3 (18)	14.3 (75)		19.3 (36)	12.5 (18)	15.1 (21)	13.5 (37)		14.6 (8)	14.4 (17)	15.4 (22)	15.7 (62)	
**Five or more times**	14.3 (3)	7.7 (4)	14.4 (17)	12.4 (65)		9.6 (18)	13.2 (19)	15.8 (22)	12.7 (35)		16.4 (9)	13.6 (16)	12.6 (18)	11.6 (46)	
**Girls**	**(n=22)**	**(n=44)**	**(n=98)**	**(n=643)**		**(n=193)**	**(n=158)**	**(n=153)**	**(n=315)**		**(n=58)**	**(n=145)**	**(n=137)**	**(n=461)**	
**Never/occasionally**	31.8 (7)	34.1 (15)	38.8 (38)	37.3 (240)	0.544	36.4 (70)	36.7 (58)	39.2 (60)	37.1 (117)	0.754	31.0 (18)	36.6 (53)	38.0 (52)	38.0 (175)	0.279
**Once or twice**	50.0 (11)	36.4 (16)	50.0 (49)	49.1 (316)		52.3 (101)	48.1 (76)	47.7 (73)	47.0 (148)		55.2 (32)	42.8 (62)	48.2 (66)	49.9 (230)	
**Three or four times**	18.2 (4)	13.6 (6)	9.2 (9)	8.9 (57)		8.3 (16)	10.1 (16)	10.5 (16)	9.5 (30)		6.9 (4)	12.4 (18)	8.8 (12)	8.9 (41)	
**Five or more times**	(0)	15.9 (9)	2.0 (2)	4.7 (30)		3.1 (6)	5.1 (8)	2.6 (8)	6.4 (20)		6.9 (4)	8.3 (12)	5.1 (7)	3.3 (15)	

Figures are presented as % (n).

* P-values were estimated using fisher’s exact tests.
